# IMPLEMENTATION OF A RISK ANALYSIS INDEX-BASED PREOPERATIVE FRAILTY SCREENING AND MANAGEMENT PROGRAM

**DOI:** 10.1093/geroni/igae098.1562

**Published:** 2024-12-31

**Authors:** Mehraneh Khalighi, Amy Thomas, Karl Brown, Katherine Ritchey

**Affiliations:** Department of Veterans Affairs Puget Sound Health Care System, Seattle, Washington, United States; VA Puget Sound Healthcare System, Seattle, Washington, United States; VA Puget Sound Healthcare System, Seattle, Washington, United States; VA Puget Sound Healthcare System, Seattle, Washington, United States

## Abstract

Frailty reduces recovery from stressors like surgery and increases postoperative morbidity and mortality. Risk Analysis Index (RAI-C) is a validated pre-surgical frailty assessment tool, however the agreement between provider-completed and patient-completed RAI is unknown. We explored the consistency and accuracy of provider-based screening and the agreement between provider-completed and patient-completed RAI-C scores. Orthopedic providers completed the RAI-C on Veterans ≥65 years of age awaiting elective total joint arthroplasty and referred patients to rehabilitative, nutritional, or geriatric specialty services based on RAI-C score. Patients were mailed the same RAI-C form to complete independently. Process measures reviewed the screening frequency and accuracy of using the referral pathway. Agreement between provider-completed and patient-completed total RAI-C score and differences within individual domains were compared. Overall, RAI screening occurred in 48% of eligible patients. Providers were less accurate in their referral of frail (RAI-C 30-36) patients than those who were robust or very frail. Screening frequency improved with dedicated nursing champions reminding providers to screen. The correlation between patient-completed and provider-completed RAI-C was moderate (r=0.634; p<0.001). Patient-completed RAI-C re-classified 29% of patients to a higher frailty category. Agreement was lowest in the domains of memory and weight loss. Preoperative frailty screening is possible with RAI-C but requires dedicated champions to improve consistency and may not yield the same result if completed by patients. Future implementation efforts should explore best practices to improve screening accuracy with the RAI-C tool by providers and patients and how referrals to preoperative services impact surgical outcomes for frail patients.

